# Estudio longitudinal de la respuesta de anticuerpos tras la vacunación contra el SARS-CoV-2 con la vacuna BNT162b2 de Pfizer-BioNtech

**DOI:** 10.1515/almed-2023-0121

**Published:** 2023-09-25

**Authors:** Leandra J. Robles Navas, Juan J. Ortega Huete, María L. Juliá Sanchis, Jorge S. Bravo Miró, Serezade Cervera Sánchez, Ricardo Molina Gasset

**Affiliations:** Residente, Servicio de Análisis Clínicos, Hospital Virgen de los Lirios, Alcoy, España; Especialista en análisis clínicos, Servicio de Análisis Clínicos, Hospital Virgen de los Lirios, Alcoy, España; Facultativa asociada, Servicio de Análisis Clínicos, Hospital Virgen de los Lirios, Alcoy, España; Enfermero, Servicio de Medicina Preventiva, Hospital Virgen de los Lirios, Alcoy, España; Enfermera, Servicio de Medicina Preventiva, Hospital Virgen de los Lirios, Alcoy, España; Jefe del Servicio de Análisis Clínicos, Hospital Virgen de los Lirios, Alcoy, España

**Keywords:** anticuerpos, COVID-19, inmunogenicidad, vacuna

## Abstract

**Objetivos:**

Se realizó un estudio para evaluar la inmunidad de trabajadores sanitarios y no sanitarios del área de salud de Alcoy (España), tras haber recibido tres dosis de la vacuna de Pfizer-BioNTech y analizar su relación con factores individuales.

**Métodos:**

Se llevó a cabo un estudio prospectivo, observacional, longitudinal, analítico, destinado a observar la inmunogenicidad en profesionales sanitarios y no sanitarios del Hospital Virgen de los Lirios, posterior a la administración de tres dosis de la vacuna de Pfizer-BioNTech. Se determinó la concentración de anticuerpos IgG frente a la proteína *spike* del SARS-CoV-2. La presencia de COVID-19 se identificó mediante detección cualitativa de anticuerpos IgG contra la proteína de la nucleocápside. Las muestras de los voluntarios se analizaron a los 15 días, 3 meses, y 6 meses de recibir la segunda dosis de la vacuna, y a los 12 meses de la tercera dosis (dosis de refuerzo). Se extrajeron datos demográficos, así como sobre factores de riesgo y el uso de fármacos inmunosupresores. Los análisis estadísticos se realizaron con el programa SPSS. Los niveles medios de anticuerpos a lo largo del tiempo se compararon mediante ANOVA.

**Resultados:**

La participación fue del 99,5 % (199/200). Las concentraciones de IgG fueron superiores en los hombres y en los sujetos obesos. No se observaron variaciones significativas con respecto a factores como el hábito tabáquico.

**Conclusiones:**

Todos los participantes desarrollaron inmunidad humoral, excepto tres sujetos, que estaban recibiendo un tratamiento inmunosupresor. Los participantes con infección previa de COVID-19 presentaron mayores niveles de anticuerpos.

## Introducción

El coronavirus de tipo 2 causante del síndrome respiratorio agudo severo (SARS-CoV-2) es un virus de la familia de *Coronaviridae* que causa la enfermedad por coronavirus de 2019 (COVID-19). Este virus fue detectado por primera vez en diciembre de 2019 en la ciudad de Wuhan, en la provincia de Hubei [1], en China. Debido al rápido incremento de casos y muertes, la Organización Mundial de la Salud declaró el brote como una pandemia el 11 de marzo de 2020 [2].

A pesar de la adopción de diversas medidas, la expansión del virus fue continua y sostenida, y su impacto a nivel mundial forzó a la comunidad científica a establecer como prioritario el desarrollo de vacunas efectivas contra este patógeno.

La proteína *spike* (S) [3] del virus se utilizó como antígeno diana, ya que este era anteriormente considerado el mejor antígeno a la hora de neutralizar los anticuerpos contra otros miembros de la misma familia. Como tal, la proteína S ha sido la principal proteína empleada para el desarrollo de las vacunas contra la COVID-19.

Las vacunas basadas en ARNm [4] presentan la ventaja de que son fáciles de producir y actúan rápidamenteuna vez se encuentran en el interior de la célula, facilitado por su acoplamiento y estabilidad en nanopartículas lipídicas encapsuladas que expresan la proteína S, lo que induce una buena respuesta humoral y celular. Este fue el método elegido por las empresas Moderna y Pfizer/BioNTech.

El objetivo de este estudio era analizar la respuesta a la vacuna de mRAN de Pfizer/BioNTech en una cohorte de profesionales sanitarios y no sanitarios, incluyendo a sujetos con y sin infección previa de COVID-19.

## Materiales y métodos

Se llevó a cabo un estudio prospectivo, observacional, longitudinal, analítico, destinado a observar la inmunogenicidad en profesionales sanitarios y no sanitarios del Hospital Virgen de los Lirios (Alcoy, provincia de Alicante, España), tras la administración de tres dosis de la vacuna de Pfizer-BioNTech. El parámetro estudiado fue la concentración de anticuerpos IgG contra la proteína S del SARS-CoV-2. Las muestras de los voluntarios se analizaron a los 15 días, 3 meses, y 6 meses de haber recibido la segunda dosis de la vacuna, y a los 12 meses de la tercera dosis (dosis de refuerzo). El objetivo del presente estudio es analizar la inmunogenicidad de la vacuna en voluntarios, a partir de dos variables principales: la edad y el sexo. Así mismo, para completar el estudio, se recopiló información sobre el índice de masa corporal (IMC), hábito tabáquico, historial médico, e infección previa de COVID-19 (y fecha en la que se produjo, en su caso), para determinar si se podían obtener otras conclusiones relevantes.

### Tamaño de la muestra

Se seleccionó aleatoriamente a 199 profesionales sanitarios y no sanitarios del área de salud de Alcoy (provincia de Alicante, España), que recibieron dos dosis de la vacuna de Pfizer-BioNTech. Los sujetos fueron estratificados en dos grupos en función de su edad (<40 años y >40 años) utilizando una hoja de Excel, donde no figuraba ningún dato sensible de los pacientes. Se seleccionó aleatoriamente a 107 sujetos del grupo etario <40 años y a 92 sujetos del grupo >40 años. Una vez seleccionados, se elaboró una lista con los números de teléfono de los sujetos seleccionados y se les notificó la posibilidad de participación en el estudio.

### Cuestionario post-vacunación

Posteriormente, los sujetos acudieron al hospital, se les proporcionó la hoja de informacióny un formulario de consentimiento informado. A aquellos que dieron su consentimiento para participar en el estudio, se les entregó una petición analitica, identificado únicamente con el número asignado al paciente, y se les citó para la extracción de la muestra sanguínea. Con el fin de preservar la confidencialidad de los datos, se les entregó un cuestionario identificado con el número que les había sido asignado. En la encuesta se recogían datos sobre la edad, el sexo, el hábito tabáquico, antecedentes de COVID-19 (en su caso), presencia de alguna patología, y uso de fármacos. Con Esta información, junto con los datos obtenidos en las pruebas analiticas, se elaboro una base de datos para su posterior análisis.

### Técnicas de determinación de anticuerpos anti-SARS- COV-2

Los niveles de anticuerpos se determinaron mediante inmunoanálisis de micropartículas quimioluminiscente (CMIA) utilizando la plataforma Alinity i^®^ de los Laboratorios Abbott (Abbott, Abbott Park, IL, EE.UU) del Servicio de Análisis Clínicos del Hospital Virgen de los Lirios Hospital de Alcoy. Se realizó la detección cuantitativa de anticuerpos IgG contra la proteína S, con una sensibilidad del 97 % y una especifidad del 99,6 %. Se consideró que los sujetos presentaban anticuerpos cuando el resultado era >49 AU/mL (unidades arbitrarias [UA]/mL).

### Análisis estadístico de los datos

Como se ha mencionado anteriormente, con la información clínica recabada y los resultados analíticos obtenidos se elaboró una base de datos, lo que permitió analizar los resultados del estudio y obtener conclusiones. El análisis estadístico se realizó utilizando el programa SPSS 15.0 (IBM Corporation, Armonk, NY, EE.UU).

Dada la muestra escogida citada anteriormente, se puede asumir normalidad en la distribución, por lo que se utilizaron test paramétricos a la hora de realizar los análisis. Se comparó la concentración media de anticuerpos a lo largo del periodo de estudio mediante análisis de varianza (ANOVA) con respecto a la edad, el sexo, el IMC, y el hábito tabáquico.

### Aprobación del Comité Ético

Para la realización del estudio se obtuvo la aprobacióndel Comité Ético de Investigación con Medicamentos (CEIM) del Hospital General Universitario de Elda (provincia de Alicante, España).

## Resultados

La participación inicial fue del 99,5 %. Un total de 107 profesionales tenían una edad <40 años, mientras que 92 profesionales tenían >40 años, con una media de edad de 42,62 años. Con respecto a los hábitos de vida, que se presumen más saludables entre los profesionales sanitarios, con una menor prevalencia de la obesidad y del hábito tabáquico, el 49 % eran no fumadores, el 54 % tenía un IMC normal, y solo el 8,5 % había sufrido infección previa de COVID-19. En la Tabla 1 se muestran las características de la población.

**Tabla 1:** Características generales de la muestra del estudio.

Variable	Media (IC 95 %)	Mínimo-Máximo
Edad, años	42,62 (38,92–42,15)	22–64
**Variables**	**Categoría**	**n (%)**
Sexo	Hombres	100 (50,3 %)
	Mujeres	99 (49,7 %)
Edad	<40 años	107 (53,8 %)
	>40 años	92 (46,2 %)
Hábito tabáquico	No fumador	96 (48,2 %)
	Ex fumador	46 (23,1 %)
	Fumador activo	57 (28,6 %)
Índice de masa corporal	Normal (18,5–24,9)	107 (53,7 %)
	Sobrepeso (25–29,9)	67 (33,6 %)
	Obesidad de clase I y II (30–40)	25 (12,5 %)

La Tabla 2 ilustra la distribución de los profesionales, existiendo una correlación entre la prevalencia de anticuerpos contra el SARS-CoV-2 (UA/mL) y la edad. Los profesionales sanitarios y no sanitarios mayores de 40 años presentaron una mayor concentración media de anticuerpos IgG que los menores de 40 años, aunque la diferencia no fue estadísticamente significativa. Los resultados obtenidos muestran que el 98,5 % de los participantes presentaban anticuerpos IgG contra la proteína S.

**Tabla 2:** Análisis de la concentración de anticuerpos IgG contra la proteína S de la COVID-19 y la edad.

Variables	Categoría edad	Concentración media de IgG, UA/mL	IC 95 %	Valorp^a^
A los 15 días de recibir la 2° dosis de la vacuna	<40 años	19,251	17,086–21,415	0,356
	>40 años	17,363	13,687–21,039	
A los 3 días de recibir la 2° dosis de la vacuna	<40 años	3,976	3,352–4,599	0,731
	>40 años	4,403	1,706–7,101	
A los 3 días de recibir la 2° dosis de la vacuna	<40 años	2,021	816–3,226	0,848
	>40 años	2,196	842–3,550	
A los 12 meses de recibir la 3^a^ dosis de la vacuna	<40 años	31,587	26,738–36,435	0,388
	>40 años	35,077	28,485–41,670	

^a^Un valor p<0,05 se consideró significativo.

La Tabla 3 muestra la distribución de los profesionales, existiendo una correlación entre la prevalencia de anticuerpos contra el SARS-CoV-2 (UA/mL) y el sexo. La concentración media de anticuerpos contra la proteína S al completar todo el ciclo de vacunación fue de 33.166.35 UA/mL (IC95 %. 29.205.16–37.127.53). A lo largo del estudio, los hombres presentaron mayor inmunidad IgG contra la proteína S que las mujeres, una diferencia que fue estadísticamente significativa a los 12 meses de recibir la tercera dosis de la vacuna.

**Tabla 3:** Análisis de la concentración de anticuerpos IgG contra la proteína S de la COVID-19 y el sexo.

Variables	Categoría sexo	Concentración media de IgG, UA/mL	IC 95 %	Valor p^a^
A los 15 días de recibir la 2° dosis de la vacuna	Hombres	18,506	15,143–21,869	0,942
	Mujeres	18,358	16,179–20,538	
A los 3 días de recibir la 2° dosis de la vacuna	Hombres	4,602	2,253–6,950	0,472
	Mujeres	3,717	3,098–4,335	
A los 6 meses de recibir la 2° dosis de la vacuna	Hombres	2,507	902–4,112	0,369
	Mujeres	1,693	873–2,512	
A los 12 meses de recibir la 3^a^ dosis de la vacuna	Hombres	39,801	33,219–46,383	0,001
	Mujeres	26,751	22,539–30,962	

^a^Un valor p<0,05 se consideró significativo.

En la Tabla 4 se puede observar la distribución de los valores de IgG contra la proteína S a lo largo del periodo de estudio, en relación con el IMC.

**Tabla 4:** Análisis de la concentración de anticuerpos IgG contra la proteína S de la COVID-19 y el IMC.

Variables	Categoría de IMC	Concentración media de IgG, UA/mL	IC 95 %	Valor p^a^
A los 15 días de recibir la 2° dosis de la vacuna	Normal	17,225	15,202–19,248	0,381
	Sobrepeso	19,345	14,634–24,055	
	Obesidad	21,161	16,424–25,898	
A los 3 meses de recibir la 2° dosis de la vacuna	Normal	3,687	3,057–4,317	0,495
	Sobrepeso	5,185	1,679–8,691	
	Obesidad	3,460	2,664–4,257	
A los 6 meses de recibir la 2° dosis de la vacuna	Normal	2,070	804–3,337	0,892
	Sobrepeso	2,327	647–4,007	
	Obesidad	1,610	160–3,059	
A los 12 meses de recibir la 3^a^ dosis de la vacuna	Normal	30,689	25,195–36,183	0,050
	Sobrepeso	32,489	26,553–38,424	
	Obesidad	45,856	31,452–60,259	

^a^Un valor p<0,05 se consideró significativo.

Aunque existieron diferencias en la generación de anticuerpos entre los participantes con un IMC normal y aquellos con sobrepeso u obesidad, estas no fueron significativas, excepto a los 12 meses de recibir la tercera dosis, cuando se alcanzó un valor p de 0,05, considerado el límite de la significación estadística. Tras realizar las pruebas *post hoc* correspondientes (Tabla 5), se observaron diferencias estadísticamente significativas entre los sujetos con un IMC normal y aquellos con obesidad.

**Tabla 5:** *Pruebas post-hoc* sobre la concentración de anticuerpos IgG contra la proteína S de la COVID-19 y el IMC.

Variable	Categoría de IMC	Concentración media de IgG, UA/mL	IC 95 %	Diferencia media	Valor p^a^
A los 12 meses de recibir la 3^a^ dosis de la vacuna	Normal	30,689	25,195–36,183	−1,780	1,000
	Sobrepeso	32,489	26,553–38,424		
	Normal	30,689	25,195–36,182	−15,167	0,050
	Obesidad	45,856	31,452–60,259		
	Sobrepeso	32,489	26,553–38,424	−13,367	1,340
	Obesidad	45,856	31,452–60,259		

^a^En las pruebas *post hoc*, se consideró significativo un valor p<0,05.

La Tabla 6 muestra la producción de anticuerpos IgG inducida por la vacuna en relación al hábito tabáquico. No se observaron diferencias estadísticamente significativas a lo largo del estudio, aunque se produjo un incremento considerable en la concentración media de IgG entre los ex fumadores, con respecto a los no fumadores y los fumadores activos.

**Tabla 6:** Análisis de la concentración de anticuerpos IgG contra la proteína S de la COVID-19 y el hábito tabáquico.

Variables	Categoría de hábito tabáquico	Concentración media de IgG, UA/mL	IC 95 %	Valor p^a^
A los 15 días de recibir la 2° dosis de la vacuna	No fumador	18,674	16,445–20,902	0,385
	Ex fumador	20,378	13,643–27,113	
	Fumador activo	16,501	13,752–19,250	
A los 3 días de recibir la 2° dosis de la vacuna	No fumador	4,077	3,348–4,806	0,239
	Ex fumador	5,854	765–10,942	
	Fumador activo	2,969	2,333–3,606	
A los 6 meses de recibir la 2° dosis de la vacuna	No fumador	2,310	877–3,743	0,446
	Ex fumador	3,173	762–5,585	
	Fumador activo	825	592–1,057	
A los 12 meses de recibir la 3^a^ dosis de la vacuna	No fumador	31,795	25,989–37,601	0,521
	Ex fumador	40,896	31,218–50,574	
	Fumador activo	28,934	22,989–34,880	

^a^Un valor p<0,05 se consideró significativo.

## Discusión

Los resultados obtenidos muestran que el 98,5 % de los participantes generaron anticuerpos IgG contra la proteína S, lo que sugiere que la vacuna confiere una elevada protección al personal de alto riesgo. Únicamente hubo tres sujetos que no generaron anticuerpos, los cuales estaban recibiendo tratamiento inmunosupresor. El tabaco ha sido señalado como un posible factor de gravedad de la COVID-19 [5, 6], aunque se han publicado pocos estudios que lo relacionen con una menor respuesta a la vacunación. En el presente estudio, los ex fumadores generaron más anticuerpos que los no fumadores y los fumadores activos, aunque la diferencia no fue estadísticamente significativa.

Existe evidencia de que los pacientes con COVID-19 con mayor IMC generan mayores niveles de anticuerpos contra el SARS-CoV-2 [7]. Pellini y col [8], que estudiaron únicamente a individuos vacunados, no hallaron ninguna relación entre el IMC y los niveles de anticuerpos IgG. Por otro lado, Watanabe y col [9] observaron que los sujetos con mayor obesidad abdominal presentaban niveles menores de anticuerpos IgG. En nuestro estudio, observamos que las concentraciones de anticuerpos solo aumentaban en los pacientes con obesidad, una vez recibidas las tres dosis de la vacuna. Son necesarios más estudios para confirmar estos hallazgos.

Dada la eficacia de las vacunas empleadas, actualmente no se recomienda comprobar la respuesta inmune midiendo los niveles de anticuerpos [9], recomendación que los resultados obtenidos en nuestro estudio respaldan. Este estudio presenta algunas fortalezas: 1) está basado en una muestra representativa de todo el personal del hospital, habiendo contado con una tasa muy alta de participación; y 2) las pruebas serológicas empleadas tienen una sensibilidad y especifidad muy altas. Algunas de las limitaciones del estudio son: 1) el tamaño de la muestra es demasiado pequeño para extraer conclusiones definitivas sobre la respuesta en la producción de anticuerpos IgG contra la proteína S tras la vacunación; 2) nuestros resultados no se pueden extrapolar a la población general, debido tanto a la edad, como al mayor riesgo de exposición a la enfermedad; y 3) dado que se trata de un nuevo virus, existen muchas cuestiones aún por dilucidar, por lo que podrían existir otros factores que podrían haber influido en los resultados que no fueron analizados. Se debería considerar realizar un seguimiento de esta cohorte de profesionales sanitarios y no sanitarios vacunados, con el fin de conocer con mayor precisión la dinámica de la inmunidad conferida por la vacuna y su persistencia a lo largo del tiempo, en vista a posteriores refuerzos de la vacuna. Todos los participantes desarrollaron una respuesta inmune humoral, excepto tres, que estaban inmunosuprimidos. Las variables relacionadas con mayores niveles de anticuerpos fueron el sexo masculino, tener un IMC >30 y haber dejado de fumar.
